# Addressing Sexuality With Older People in Primary Care and Hospitals: A Systematic Review of Professional Perceptions and Experiences

**DOI:** 10.1155/jare/9289942

**Published:** 2026-07-02

**Authors:** Ana Carolina Frias, Anabela Mota-Pinto, Cristina C. Vieira, Lélita Santos

**Affiliations:** ^1^ Polytechnic University of Coimbra, Coimbra Education School, Coimbra, Portugal; ^2^ InED–Centre for Research and Innovation in Education, Polytechnic University of Coimbra, Coimbra, Portugal; ^3^ CIDTFF–Research Centre on Didactics and Technology in the Education of Trainers, University of Aveiro, Aveiro, Portugal, ua.pt; ^4^ Faculty of Medicine, University of Coimbra, Coimbra, Portugal, uc.pt; ^5^ ICBR (Coimbra Institute for Clinical and Biomedical Research), Coimbra, Portugal; ^6^ CIMAGO (Centre for Research in Environment, Genetics and Oncobiology), Faculty of Medicine, University of Coimbra, Coimbra, Portugal, uc.pt; ^7^ Faculty of Psychology and Educational Sciences, University of Coimbra, Coimbra, Portugal, uc.pt; ^8^ Adult Education and Community Intervention Research Centre (CEAD), University of the Algarve, Faro, Portugal; ^9^ Local Health Unit of Coimbra, Coimbra, Portugal

**Keywords:** aging, primary health care, sexual health

## Abstract

Sexuality in older age remains a taboo subject. Little is known about the perceptions and experiences of healthcare professionals when addressing this topic with older adults. This systematic review, registered in PROSPERO, aims to understand the perceptions, practices, and recommendations of healthcare professionals in primary and hospital care regarding the approach to sexuality and sexual health in older people. The 17 selected studies, conducted in six countries, show that physicians and nurses rarely discuss sexuality with their older patients, often assuming that they are not interested in the topic, but also due to a lack of time and willingness to address it. The studies recommend both initial and continuing education on sexuality and aging, as well as the development of protocols for assessing the sexual health of older people in healthcare settings. The absence of studies from Portugal and the passive role of professionals in promoting the sexual health of older people further reinforce the need for research in this area.

## 1. Introduction

Sexuality is a central dimension of human development, encompassing sex, gender identities and roles, sexual orientation, eroticism, pleasure, intimacy, and reproduction, and is experienced in a unique way throughout the life cycle [1]. However, sociocultural values continue to delegitimize the sexuality of older people, who are often perceived as asexual, which undermines the recognition and promotion of their sexual rights [2–4].

This invisibility is also reflected among older adults themselves, who often present low levels of sexual health literacy, reductive views, and attitudes marked by prejudice [5, 6]. Nevertheless, research shows that sexuality remains relevant in older age and that positive sexual attitudes and experiences are associated with higher self‐esteem, quality of life, and overall well‐being [7]. Moving away from hegemonic perspectives centered on decline, older adults may experience positive changes in their sexual lives, attributing importance to reciprocity of desire, dating, emotional intimacy, and values such as love, companionship, and closeness [8, 9].

Although age does not constitute a barrier to experiencing sexuality, the aging process may involve physiological changes that impact sexual health, influenced by biological, psychological, relational, and social factors, which require appropriate assessment and monitoring [7]. The adoption of a positive approach to sexuality, grounded in human and sexual rights, is essential to ensure equality, privacy, sexual education, pleasure, and the right to safe sexual experiences free from coercion and discrimination [10].

Health services must therefore position themselves as safe and inclusive spaces that recognize and value the sexual dimension of aging, moving beyond a purely pathological perspective and promoting access to comprehensive and scientifically grounded information [9, 11]. However, the scarcity of evidence on the interaction between healthcare professionals and older adults regarding sexuality, as well as difficulties in accessing appropriate guidance and dissatisfaction with the care received, reveals significant gaps. It is therefore essential to further investigate healthcare professionals’ perceptions and experiences in order to identify best practices, barriers, and facilitators for promoting sexual health in this population [3, 6, 12, 13].

## 2. Methods

This systematic review was conducted in accordance with the guidelines of the Joanna Briggs Institute (JBI), following the protocol registered in the PROSPERO platform (registration number CRD420251008998).

### 2.1. Objectives of the Review

General Objective: To understand the perceptions, practices, and recommendations of health professionals in primary health care (PHC) and hospital settings regarding the approach to sexuality and sexual health in older adults.

### 2.2. Specific Objectives

• Identify physicians’ and nurses’ attitudes and perceptions regarding addressing sexuality in older adults;
• Analyze how frequently physicians and nurses discuss sexuality and sexual health with older adults;
• Identify recommendations for promoting sexual health as perceived by physicians and nurses in PHC and hospital services, as well as for public policies in the field.

### 2.3. Search Strategy

The systematic review began with the formulation of the guiding question according to the PICoD strategy (Table 1): What are the perceptions, practices, and recommendations of health professionals regarding the approach to sexuality and sexual health in older adults in PHC and hospital settings?

**TABLE 1:** PICOD strategy and study eligibility criteria.

Acronym meaning		Component of the question in the study review	Eligibility criteria	
			Inclusion	Exclusion
P	Population	Health professionals (doctors and nurses)	‐ Studies with doctors and nurses who take care of elderly people ‐ Studies that include, in addition to professionals, users over 65 years of age	‐ Studies with informal caregivers and/or with patients under 65 years old, and/or with dementia.

I	Intervention	Approach to sexuality and sexual health with older people	‐ Studies in which the central theme is sexuality or sexual health, or about the promotion of health or sexual education; education about the prevention of sexually transmitted infections in the elderly; interventions and strategies of communication about sexuality with the elderly; health assessment of older people; gerontogeriatric care.	‐ Studies that approach specifically medical interventions within the scope of a disease; ‐ Studies about health care only directed to LGBTQIA + people.

C	Context	Primary Health Care (PHC) Units and Hospital Health Care Units	‐ Studies realized in hospitals (In any inpatient service, medical consultations of any specialty, or day hospital) and PHC, public or private, in every country.	‐ Studies realized in residential structures for the elderly or in home care.
o	Outcomes	Identification of the perceptions, attitudes, knowledge, and practices of health professionals about sexuality and promotion of sexual health in older people.		
D	Design/study design	Qualitative and quantitative primary studies	‐ Qualitative and quantitative primary studies.	‐ Randomized studies, systematic reviews, or scale validation.

The search was conducted in April 2025 in Scopus, PubMed, LILACS, EBSCO (CINAHL via EBSCO), and RCAAP, following the eligibility criteria (Table 1). Validated Medical Subject Headings (MeSH) descriptors were used: Sexuality; Sexual Activity; Primary Health Care; Health Care, Primary; Primary Care; Family Physicians; Primary Care Nursing; Sexual Health; Sex Education; Aged; Elderly; Oldest Old; Aged, 80 and over; Attitude of Health Personnel; Early Intervention; and Educational Health Personnel. The terms were combined using the Boolean operators “OR” and “AND.” Full‐text, open‐access articles published between 2015 and 2025 in English, Spanish, and Portuguese were included.

Study selection was performed by two independent researchers (Ana Carolina Frias and Anabela Mota‐Pinto) and reported according to the Preferred Reporting Items for Systematic Reviews and Meta‐Analyses (PRISMA) guidelines. Methodological quality and risk of bias were independently assessed using the JBI critical appraisal tools. Any discrepancies were discussed and resolved by consensus. After full‐text review, relevant data were extracted and synthesized in a summary table.

The exclusive inclusion of open‐access articles was intended to ensure full accessibility to all sources used, promoting transparency, the democratization of knowledge, and enabling both the scientific community and practitioners to verify the findings. It also aimed to facilitate the translation of knowledge into practice, particularly in contexts with limited access to subscription‐based scientific databases. However, it is acknowledged that this decision may have introduced a potential selection bias, thereby limiting the comprehensiveness and representativeness of the evidence considered.

## 3. Results

**FIGURE 1:**
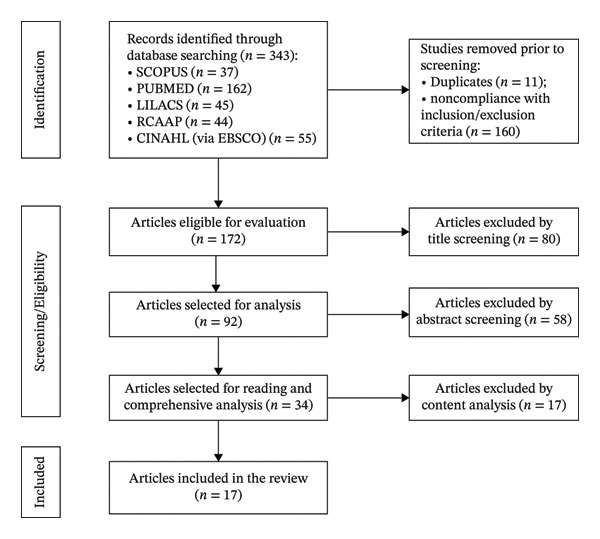
Flowchart of the study selection process according to the PRISMA diagram.

From the 343 identified studies, 17 were included in the systematic review, as shown in the PRISMA flow diagram (Figure 1 below).

The included articles were assessed for methodological quality and level of evidence according to the JBI criteria [14]. Of the 17 studies, four were classified as analytical cross‐sectional studies (JBI Level IV.B) and appraised using the JBI Critical Appraisal Checklist for Analytical Cross‐Sectional Studies, while the remaining 13 were qualitative studies (Level 3—single qualitative study: meaningfulness) and assessed using the JBI Critical Appraisal Checklist for Qualitative Research. The results were subsequently analyzed and synthesized (Table 2).

**TABLE 2:** Data extraction synthesis.

Study (S)	Aim	Participants and context	Results and conclusions Conceptions and practices of health professionals	Level of evidence and quality appraisal Score
[15] (S_1_)	To assess the knowledge and attitudes of Family Health Strategy nurses regarding sexuality in old age.	56 nurses from the Family Health Strategy, in the urban area of Ceará, Brazil	‐ The majority of nurses (94.64%) reported knowing how to guide older adults who inquire about sexual health, and 60.71% reported addressing this topic in their nursing consultations. ‐ Based on the application of the ASKAS scale, there is an overall adequate understanding of sexuality in older age; however, conservative attitudes persist, particularly in relation to the recognition of sexual interest among older adults and the acceptance of masturbation.	JBI Level IV:B Quality Appraisal Score: 8/8 (100%)

[16] (S_2_)	Determine how often doctors discuss sexuality with their elderly patients with chronic pain.	155 hospital doctors from different specialities (except urology and gynaecology) who treat elderly patients with chronic pain. São Paulo, Brazil.	‐ Physicians frequently manage patients aged 70–79 years presenting with nociceptive pain. ‐ Although 87% consider older adults to be sexually active, 63% do not routinely address sexuality during consultations. The main reasons for not addressing this topic include lack of time, fear of embarrassing the patient, perceived lack of competence, and the belief that it falls under the responsibility of another physician. ‐ Physicians with more years of professional experience are more likely to discuss sexuality with older patients.	JBI Level IV:B Quality Appraisal Score: 8/8 (100%)

[17] (S_3_)	Assessing family doctors’ perceptions of sexuality in older adults.	16 family doctors from primary health care units (urban and rural). Israel.	‐ Physicians do not routinely ask older adults about sexuality (including intimacy, marital relationships, and related aspects) during routine consultations. ‐ The reasons for not addressing this topic include heavy workloads, lack of time, the perception that it is not a priority in the care of this population, and fear of offending patients or damaging the physician–patient relationship. ‐ Discussions about sexuality with older patients tend to occur when physicians feel comfortable doing so or in cases of erectile dysfunction. In such situations, the focus is mainly on sexual functioning and pharmacological treatment options, although the psychological dimension is sometimes acknowledged as needing greater attention. Clinical approaches are predominantly physiological, emphasizing symptoms, disorder prevalence, medication, and comorbidities.	JBI Level 3. (Meaningfulness). Single qualitative study. Quality Appraisal Score: 8/10 (80%)
[18] (S_4_)	Analyse doctors’ perspectives on sexuality in older people.	38 doctors (specialising in gynaecology, urology or family medicine, or certified in human sexuality by the Israeli Society for Sexual Therapy (ISST) in Israel.	‐ The majority of physicians report that sexuality is important at all ages and that they are willing to address this topic in their medical consultations. ‐ In clinical encounters, older adults are often assumed to be heterosexual, whereas in younger adults, sexual orientation is explored more explicitly and in a way that embraces diversity. In general, physicians perceive that older men and women have different concerns and expectations, with men more frequently associated with the importance of penetrative sex and women with a greater emphasis on intimacy and emotional closeness. ‐ In consultations, when addressing sexual dysfunction in older adults, physicians tend to adopt a predominantly biomedical approach, in contrast to the broader biopsychosocial approach typically used with younger adults. Pharmacological treatment is commonly prescribed for older patients, usually without referral for psychotherapy.	JBI Level 3. (Meaningfulness). Single qualitative study. Quality Appraisal Score: 8/10 (80%)

[19] (S_5_)	Explore attitudes and perspectives of doctors from primary health care about sexual health in middle‐age adults and the elderly	35 primary healthcare physicians (public and private facilities) from Trinidad and Tobago.	Physicians report several barriers when addressing sexual health with older adults, related to professionals, patients, and contextual factors. ‐ Professional‐related barriers include avoiding the use of terms such as “sex,” “sexuality,” or “reproductive organs”; a greater likelihood of discussing sexuality with patients of the same ethnicity; insufficient training on sexual health in adulthood within medical curricula; and the perception that sexual history is relevant but not a priority in consultations, which typically only occurs when the patient initiates the discussion. ‐ Patient‐related barriers include older adults’ preference for discussing sexuality with professionals of the same sex and the perception that a large age gap between physician and patient makes such discussions inappropriate or disrespectful. ‐ Other contextual factors include paternalistic models of care, lack of human and time resources, absence of sexual health policies, lack of privacy in primary healthcare settings, and lower utilization of primary care services by men.	JBI Level 3. (Meaningfulness). Single qualitative study. Quality Appraisal Score: 9/10 (90%)

[20] (S_6_)	Describe the actions and knowledge of the nursing team regarding the prevention and diagnosis of HIV and AIDS in elderly people in PHC.	10 health professionals (3 nurses and 7 nursing professionals), in a Basic Health Unit, in a metropolitan region of Brazil.	Nurses’ practices and perceptions: ‐ Nurses often do not consider older adults as potentially sexually active and tend to assume a lack of interest in sexual health topics, including HIV prevention. ‐ There is limited knowledge of current clinical protocols for HIV diagnosis and management (e.g., partner notification, referral pathways, and reporting procedures following a positive test result), and nurses generally do not receive or actively seek training in this area. ‐ Although HIV testing is performed, condoms are rarely distributed to older adults. ‐ There is a lack of health education and HIV prevention activities targeted at older adults, both in clinical consultations and in community or group settings, and informational materials displayed in healthcare facilities are not actively discussed with patients. ‐ Nurses report difficulties in discussing sexuality, particularly with older adults.	JBI Level 3. (Meaningfulness). Single qualitative study. Quality Appraisal Score: 9/10 (90%)

[21] (S_7_)	To identify differences and similarities in the perception of healthcare providers in Colombia regarding sexuality and sexual health in older adults. To analyse experiences in addressing sexuality with older adults.	19 professionals (doctors, nurses, psychologists) from institutions specializing in sexual and reproductive health. Colombia (Bogotá, Barranquilla, Cali, Medellín).	Healthcare professionals’ perceptions, challenges, facilitators, and recommendations: ‐ Healthcare professionals recognize benefits in addressing sexual health with older adults, particularly in relation to the management of comorbidities. ‐ Challenges in addressing sexuality during consultations include older women’s tendency to adopt a more reserved attitude and avoid discussing sexual health, gender discordance between patient and professional, and the difficulty experienced by patients who have been exposed to family violence in initiating conversations about sexuality. ‐ Facilitating factors identified by professionals include working in institutions specialized in sexual and reproductive health, which enhances their capacity to listen to patients; adopting a more positive perspective on sexuality and recognizing older adults as sexual beings; and having flexibility to adapt consultations to patients’ concerns as they arise. ‐ Professional recommendations include optimizing the consultation space to ensure older adults are informed about their sexual rights; encouraging reflection on sexuality and the body; and integrating older adults into health programs that promote the visibility of sexuality in later life.	JBI Level 3. (Meaningfulness). Single qualitative study. Quality Appraisal Score: 10/10 (100%)

[22] (S_8_)	To understand the experiences of older adults and healthcare professionals regarding sexuality in older adults.	23 healthcare professionals and 12 elderly individuals in Primary Healthcare. Paraíba, Brazil.	‐ Although healthcare professionals report having knowledge about sexuality, including its subjective dimension beyond purely biophysiological aspects, they do not consistently apply this knowledge in clinical practice, particularly with older adults. Care is often driven by patients’ complaints and framed within a predominantly biomedical and curative perspective. Professionals also report that the topic is frequently not addressed due to forgetfulness and lack of time. ‐ Older adults demonstrate a reductive view of sexuality, often limited to marriage and reproductive sexual activity. They tend to associate condom use exclusively with pregnancy prevention and consider it unnecessary in older age.	JBI Level 3. (Meaningfulness). Single qualitative study. Quality Appraisal Score: 9/10 (90%)

[23] (S_9_)	To understand the academic training of geriatricians regarding sexuality and aging, as well as their practical approach to the subject in their consultations.	12 geriatricians (9 supervisors and 3 interns) from the Geriatrics department of a public hospital in Recife, Brazil.	‐ Physicians understand sexuality as a broad, multidimensional concept that is present throughout the life course and should be addressed with older adults. However: They do not consistently consider its social dimension or the specificities of sexuality across the life cycle, often placing greater emphasis on sexual relationships. The topic is usually not addressed during medical consultations, and healthcare professionals often adopt a passive stance, waiting for patients to initiate the discussion. Physicians perceive resistance from older adults when discussing sexual health in healthcare settings. Although they consider it the physician’s responsibility to initiate such conversations, sexuality is not routinely included in comprehensive geriatric assessments and is often left to the individual discretion of clinicians. ‐ Barriers to addressing sexuality in geriatric consultations include: the presence of family members during consultations; fear of causing embarrassment; lack of training and preparation to address personal taboos and prejudices; limited research on the topic; and the absence of standardized protocols for assessing sexuality in older adults.	JBI Level 3. (Meaningfulness). Single qualitative study. Quality Appraisal Score: 9/10 (90%)

[24] (S_10_)	To analyse the performance of healthcare professionals in providing care to elderly individuals diagnosed with HIV and AIDS in a public health service.	9 healthcare professionals (doctors, nurses, pharmacist), psychologist, and social worker. Public service providing specialized care for sexually transmitted infections. Minas Gerais, Brazil.	Healthcare professionals report discomfort when discussing sexuality and sexual activity with older patients. ‐ In the care of older adults living with HIV, high workload is a limiting factor in addressing sexuality and sexual practices. ‐ Some professionals working in specialized HIV care services are surprised to encounter older adults living with HIV, due to the assumption that this population is not sexually active.	JBI Level 3. (Meaningfulness). Single qualitative study. Quality Appraisal Score: 9/10 (90%)

[25] (S_11_)	To investigate, in elderly people with HIV/AIDS and healthcare professionals, the reasons for late diagnosis of HIV infection in the elderly.	23 healthcare professionals (11 nurses, 12 doctors) and 11 elderly individuals. Specialized Infectious Disease Outpatient Service and Family Health Units. São Paulo, Brazil.	Healthcare professionals’ perspectives: ‐ Physicians and nurses do not routinely inquire about older patients’ sexual lives, often due to age differences and gender discordance between clinician and patient. Sexual health is typically addressed only after an HIV diagnosis, primarily for the purpose of providing information on infection prevention and transmission. ‐ In primary healthcare settings, HIV testing is not routinely requested due to the lack of clear clinical guidelines. Some professionals suggest that HIV testing should be conducted during public health prevention campaigns, targeting specific groups such as widowed older adults, individuals who use drugs, and those with multiple sexual partners, while older adults in stable relationships are often not considered for testing. ‐ In older adults presenting signs and symptoms suggestive of HIV infection, other pathologies are often investigated first.	JBI Level 3. (Meaningfulness). Single qualitative study. Quality Appraisal Score: 10/10 (100%)

[26] (S_12_)	To analyse the association between sociodemographic, occupational, and work practice variables of nurses at different levels of care on their knowledge and attitudes about the sexuality of older adults.	221 nursing professionals. Healthcare institutions within the Unified Health System, Brazil.	Nurses demonstrate a good level of knowledge about sexuality in older adults, as well as generally tolerant and permissive attitudes. However, they do not routinely address this topic with patients, nor do they discuss preventive or diagnostic measures. The majority report not feeling adequately prepared to address sexuality with older adults and having never participated in training on this subject. Physicians with greater knowledge of the topic tend to adopt a more positive and confident attitude when addressing sexuality with patients. The age of healthcare professionals also influences whether they address sexuality, with older professionals reporting greater comfort in initiating such conversations.	JBI Level IV:B Quality Appraisal Score: 8/8 (100%)

[27] (S_13_)	To analyse the perspectives of primary healthcare physicians, with and without training in human sexuality, regarding discussions of sexuality with their elderly patients.	38 primary healthcare physicians (17 without specific training in human sexuality; 21 certified as sex therapists) in Israel.	Physicians without prior training in human sexuality do not routinely ask older patients about sexual functioning or sexual problems, citing workload, lack of time, and fear of offending patients as main constraints. When sexual issues are addressed, they tend to be interpreted from a predominantly physiological perspective. Male sexual dysfunction is often considered common and easily treated with medication, whereas female sexual problems are viewed as more complex and requiring a comprehensive approach involving both medical and emotional dimensions. Physicians with prior training in human sexuality are more likely to establish open communication with older adults. They consider intimate and marital relationships as an integral part of clinical assessment in their daily practice and adopt a biopsychosocial perspective when diagnosing sexual problems, including medical, social, relational (dyadic), and psychological dimensions. Although they may offer a broader range of medication‐based interventions compared to physicians without such training, they emphasize the importance of psychological and relational aspects of sexual activity. Training in human sexuality appears to enhance physicians’ comfort in addressing not only the physiological aspects of patients’ concerns but also their psychosocial dimensions, promoting greater sensitivity and confidence in clinical practice.	JBI Level 3. (Meaningfulness). Single qualitative study. Quality Appraisal Score: 9/10 (90%)

[28] (S_14_)	To analyse factors at the individual and structural levels that potentially facilitate discussions about sexuality between doctors and older people.	15 doctors (specializing in gynaecology, family medicine, psychiatry, urology, and rehabilitation) with certified training in human sexuality in Israel.	From the perspective of respondents with training in sexuality: Physicians are less likely to discuss sexuality with patients over 75 years of age than with younger patients. In general, they assume that this topic is not particularly relevant in the lives of older adults. Although they recognize their responsibility to support older people in addressing sexual concerns, they are not always fully aware of these needs. Medical education does not adequately cover sexuality in older age or provide sufficient communication strategies for discussing and assessing this topic in clinical practice. Older adults also tend to find it more difficult to initiate conversations about sexuality with physicians compared to younger patients. Recommendations: Open communication about sexuality (including desires, needs, and sexual dysfunctions) is necessary not only in clinical consultations but also in media and health policy contexts. Physicians should prioritize active listening to older adults rather than focusing on having complete knowledge about sexuality. Before initiating discussions about sexuality with older patients, physicians should establish a trusting therapeutic relationship. It is also necessary to raise awareness of sexuality in older age in a non‐stigmatizing way; to enhance the knowledge of medical students and physicians; to provide accurate and up‐to‐date information to older adults; and to promote greater openness in clinical consultations, creating a welcoming environment in which older patients feel comfortable discussing their sexual health needs.	JBI Level 3. (Meaningfulness). Single qualitative study. Quality Appraisal Score: 9/10 (90%)

[29] (S_15_)	To analyse the professional practice of physicians and nurses in the Family Health Strategy regarding the sexuality of the elderly.	12 health professionals (6 doctors, 6 nurses) from Family Health Units, Ceará, Brazil.	Physicians and nurses working in Family Health Units recognize the importance of addressing sexuality with older adults, particularly in light of the increasing incidence of HIV and AIDS. However, they report difficulties in addressing this topic during consultations due to fear of causing discomfort, especially among male patients. Healthcare professionals tend to wait for patients to present sexual health complaints before addressing the topic. When older adults report issues related to menopause or erectile dysfunction, they are usually referred to specialized care.	JBI Level 3. (Meaningfulness). Single qualitative study. Quality Appraisal Score: 10/10 (100%)

[30] (S_16_)	Understanding the practices of primary healthcare physicians in discussing sexual health with older adults.	37 doctors at a hospital in Ontario, Canada.	Approximately 54% of respondents reported having adequate knowledge to discuss and manage sexual health issues in older age. Physicians are more likely to address sexual health with patients aged 50 to 75 than with those over 75, both among men (*p* < 0.0001) and women (*p* < 0.0001). In male patients, discussions typically focus on erectile dysfunction and sexually transmitted infections, whereas in female patients they more commonly address atrophic vaginitis, bleeding, and dyspareunia. Factors limiting these discussions include lack of time, patients’ multiple comorbidities, and the perceived lack of interest in sexual activity among older adults.	JBI Level IV:B Quality Appraisal Score: 7/8 (87,5%)

[31] (S_17_)	To understand the perspectives of patients and physicians regarding HIV testing in old age; To identify clinical implications and improvement interventions in the application of the test.	20 doctors from various specialties and 20 patients aged 50+, diagnosed late with HIV. HIV Care Centres. England	Physicians’ perspectives and practices: Physicians acknowledge difficulties in addressing sexuality with older adults and report perceiving that older patients would find it offensive to be offered HIV screening tests. They also report discomfort when discussing HIV with older adults and generally do not address sexuality with this population due to a lack of knowledge of appropriate communication strategies. There is a tendency to assume a priori that older adults are at low risk of HIV infection, based on the perception that they are more responsible than younger individuals and therefore less likely to engage in risky behaviours, reflecting an inaccurate risk perception. In some cases, HIV symptoms are misinterpreted and attributed to aging. Best practices to minimize late diagnosis include: Offering HIV testing routinely, regardless of age; Improving knowledge among physicians and older adults through tailored information and health promotion materials; Designing personalized HIV testing services for older adults, particularly within primary healthcare settings.	JBI Level 3. (Meaningfulness). Single qualitative study. Quality Appraisal Score: 10/10 (100%)

The included studies were conducted in Brazil _(S1, S2, S6, S8, S9, S10, S11, S12, S15)_, Colombia _(S7)_, Israel _(S3, S4, S13, S14)_, Trinidad and Tobago _(S5)_, Canada _(S16)_, and England _(S17)_. They took place mainly in primary healthcare or clinics, and only three in hospitals _(S2, S9, S16)_. Participants included physicians (*n* = 9), nurses (*n* = 4), and multidisciplinary teams composed of physicians, nurses, and other health professionals (*n* = 4). They used interviews (*n* = 13), questionnaires (*n* = 2), and the Aging Sexual Knowledge and Attitudes Scale (ASKAS) (*n* = 2). The findings allowed the identification of three main themes: (1) attitudes and perceptions regarding sexuality and the promotion of sexual health; (2) barriers to discussing sexuality; and (3) facilitating factors and recommendations for practice.

### 3.1. Attitudes and Perceptions Regarding Sexuality and the Promotion of Sexual Health

In several countries, physicians and nurses rarely discuss sexuality with older adults, demonstrating limited attention to this dimension of health, similar to what is observed in the provision of care to adults in general [32]. They often assume that the topic is not relevant to this population _(S3, S8, S11, S12, S13, S14, S17)_, based on the belief that older adults are not sexually active and lack sexual interest (_S5, S6, S10, S16_).

During consultations, healthcare professionals rarely use terms such as “sex,” “sexuality,” or “reproductive organs.” They also report difficulty in recognizing sexual diversity among older adults, including non‐heterosexual identities (_S4_) and practices such as masturbation (_S1_). These realities, often overlooked by professionals, contribute to discomfort and reinforce heteronormative assumptions, which may hinder communication and the therapeutic relationship, particularly when addressing diverse gender identities [33].

Healthcare professionals often exhibit ageist attitudes and beliefs, grounded in a view of aging as a process characterized by decline, disease, and diminished sexuality [4]. They demonstrate a limited biopsychosocial understanding of sexuality as a dimension of affective relationships and overall quality of life in older adults [3, 12]. Only physicians with prior training in sexuality recognized the importance of this dimension across the life course, engaged in open communication with older adults about intimate and marital relationships, and identified sexual concerns from a medical, social, and psychological perspective (_S4, S13_).

Sexual health promotion interventions often reflect a passive stance among healthcare professionals, stemming from a lack of training in addressing the topic, as well as from personal beliefs, institutional constraints, and the predominance of the biomedical model. In general, professionals tend to address sexual health only when prompted by patients presenting related concerns. In such cases, discussions are typically limited to the prevention and treatment of sexually transmitted infections (_S5, S6, S11, S16, S17_), as well as to symptoms and pharmacological management of conditions such as erectile dysfunction and premature ejaculation in men, and menopause, vaginismus, and decreased sexual desire in women (_S3, S4, S5, S7, S8, S9, S13, S15, S16_). Conceptions of sexuality and aging are often centered on physical and biological aspects of sexual function (_S3, S4_), reinforcing a genital‐focused perspective and prioritizing the prevention and identification of pathologies related to sexual activity [12]. A medicalized discourse predominates, assuming that individuals seeking healthcare primarily due to illness are mainly interested in treatment, thereby framing interventions as predominantly therapeutic and overlooking the broader impact of illness on quality of life and psychological well‐being [11].

Physicians identify different concerns between men and women—men often focus on penetrative sex, while women emphasize emotional closeness—but they rarely explore the psychological and social dimensions shaped by gendered socialization. This contributes to the reinforcement of stereotypes that may hinder a healthy experience of sexuality in older age for both women and men (_S4_). In cases of sexual dysfunction, physicians tend to prescribe medication without addressing psychosocial factors or referring patients for psychotherapy, in contrast to their approach with younger adults (_S4_). During consultations, they do not routinely conduct proactive assessments of patients’ knowledge regarding sexual health and safe sex practices, despite their importance, for example, in the prevention of sexually transmitted infections, which are increasing among older adults [5]. Furthermore, discussions about sexuality with older adults often occur only after a positive HIV diagnosis (_S11_). Although nurses carry out HIV awareness activities for the general population, such as distributing posters and leaflets, they rarely provide condoms to older adults (_S6_). The recommendation for testing often depends on professionals’ perceptions, who typically do not suggest it for older adults due to a perceived lower risk (_S17_), or only recommend it during public health campaigns, particularly for older widowed men, people who use drugs, or those with multiple partners, thereby excluding individuals in stable relationships (_S11_).

The difficulty healthcare professionals experience in establishing clear and direct communication about sexuality highlights the need for health‐promoting interventions, even in the context of illness, as well as for the deconstruction of myths that may prevent older men and women from, for example, acknowledging and experiencing sexual pleasure without feelings of guilt or shame [6, 34]. Although these aspects are not consistently reflected in current practices, it is important to assess potential ageist beliefs and attitudes held by older adults regarding themselves and their sexuality, avoiding their normalization [2]. It is also essential to use educational resources that address intimate relationships [13] and to promote discussions that go beyond dysfunction and pathology, in order to provide comprehensive and empowering sexuality education [10]. Such approaches support informed decision‐making and help uncover silences surrounding possible situations of violence or sexual abuse [36].

### 3.2. Barriers to Discussing Sexuality

The main barriers identified include lack of time due to heavy workloads and insufficient human resources (_S2, S3, S5, S8, S10, S13, S16_); fear of embarrassing, offending, or causing discomfort (_S2, S3, S5, S9, S13, S15, S17_); discomfort resulting from insufficient training on the topic (_S2, S3, S5, S6, S9, S12, S14, S17_); lack of concordance regarding ethnicity (_S5_) and gender (_S5, S7, S11_); significant age differences between professionals and patients (_S5, S11, S12_); the perception that sexuality is not a priority when caring for older adults with comorbidities (_S3, S5, S16_); and the belief that addressing sexuality falls under the responsibility of another medical specialty (_S2_).

Addressing this topic requires healthcare professionals to feel comfortable, possess adequate knowledge and experience, and, above all, engage in self‐reflection regarding their attitudes and values related to sexuality [3]. Although some professionals consider themselves knowledgeable, many report lacking the training and skills needed to discuss sexuality with older adults in a comprehensive manner, encompassing physical, emotional, and mental health, as well as beliefs and attitudes [32].

In Portugal, the training of physicians and nurses shows limited inclusion of sexuality in curricula, largely shaped by the dominance of heteronormative and cisnormative perspectives, which do not adequately prepare professionals to address this topic and may contribute to the perpetuation of stereotypes within the population [33]. This training should incorporate a gender perspective within an intersectional framework, promoting critical reflection on multiple forms of inequality, including ageism, sexism, and racism, as well as on the stereotypes and beliefs that permeate everyday practice [20]. It is essential to clarify the use of inclusive language and strategies for its application in clinical encounters, as these facilitate the development of a respectful and supportive therapeutic environment [33]. Additionally, professionals should be encouraged to establish clear and accessible communication, avoiding overly technical or obscure terminology that may hinder understanding, while also being attentive to internalized feelings of shame, particularly among certain groups of older adults, which can act as a barrier to discussing sexuality with healthcare providers. Furthermore, training should address aspects such as availability, empathy, and interest, as well as strategies for assessing sexual health and enhancing communication. This is particularly relevant in a context where care is increasingly described as dehumanized, characterized by a weakening of the therapeutic relationship [11].

Healthcare professionals often do not consider PHC settings to be appropriate or privileged environments for addressing sexuality with older adults, citing barriers such as lack of privacy, paternalistic models of care, and the presence of family members during consultations (_S9_), which may inhibit discussions of intimacy (_S5_). Additional challenges include patients’ difficulty and resistance in discussing the topic (_S6, S7, S8, S9, S14, S16_), often associating sexuality primarily with sexual activity and reproduction (_S8_), as well as gender‐related patterns, such as men seeking PHC services less frequently (_S5_) and women being more reserved (_S7_). Although older adults consider primary care physicians an important source of support for addressing sexual health, they tend to seek help only when experiencing sexual problems [13]. They may also feel ashamed due to fears of being perceived as inappropriate or obscene, while simultaneously experiencing discrimination related to this dimension, which remains socially misunderstood [7, 8]. These barriers hinder the recognition of sexual health needs, limit understanding of potential interactions between sexuality and chronic conditions, and reduce opportunities for counseling that could support the quality and harmony of intimate relationships in older adults [3, 7, 34].

### 3.3. Facilitating Criteria and Recommendations for Practices

Healthcare professionals consider initial and continuing training in sexuality education to be essential for the provision of care (_S2, S5, S6, S12, S13, S14, S17_), consistent with findings in the literature [3, 7, 32]. Additionally, factors such as increased human resources (_S5_), the implementation of sexual health assessment protocols (_S5, S6, S9_), longer professional experience (_S2_), and experience in specialized sexual health services appear to facilitate effective communication and a more positive approach to sexuality with older adults (_S7_).

Although there are limited studies on effective approaches to addressing sexuality in older adults, it is recommended that physiological, sociocultural, health (not limited to physical health), relationship, and sexual health knowledge factors be systematically assessed [3, 7]. The PLISSIT model, developed by Annon in 1976, represents a feasible framework for implementation by physicians and nurses [34]. According to this model, the professional first seeks permission (P) to address sexual health through questions that encourage patients to express their sexual concerns; then provides limited information (LI) regarding normal and pathological changes that may affect sexual health, thereby addressing misconceptions; offers specific suggestions (SS) tailored to individual needs; and, when necessary, proceeds to intensive therapy (IT) or referral [34]. The use of validated instruments to assess sexual health knowledge is also recommended, particularly in the context of HIV and AIDS prevention among older adults, where condom use remains low and systematic sexual health education interventions are lacking [5].

Systematic and multidisciplinary approaches to sexuality are needed, centered on the individual and addressing motivations or lack thereof for sexual activity, the subjectivity of the sexual and sensual self, self‐image, beliefs, values, disposition, mood, and health conditions, while moving beyond perspectives restricted to sexual activity and vaginal intercourse [3, 7, 34]. In addition, healthcare professionals should recognize that older adults living with chronic illnesses also wish for sexuality to be included in routine care and prefer that such discussions be initiated by physicians and nurses [38].

The studies analyzed indicate the need to educate older adults about sexual rights; provide accurate information without assuming complete knowledge of the subject; integrate them into sexual health programs; ensure a welcoming environment where they can openly express their needs and concerns; and involve media and health policies in promoting a positive approach to sexuality and aging (_S7; S14_).

Although not explicitly mentioned in the included studies, it is also important to address sexual pleasure [35, 36], including discussions on condom use and its dual protection function [39]. A comprehensive sexual health assessment should include sexual history of diseases, infections, and other relevant medical conditions; medication use; sexual attitudes and relationships; history of sexual abuse; sexual and gender orientation; and identification of risk and preventive factors [11].

Promoting sexual health in older age requires institutional recognition of this dimension as an integral component of healthy aging, free from stigma and misinformation, and one that acknowledges diversity related to gender, sexual orientation, marital status, functional status, and life experiences, all of which influence how sexuality is experienced and expressed. A policy on the sexual rights of older adults is needed to prevent discrimination and its associated health consequences [13]. This should be accompanied by a shift in societal attitudes toward aging, ensuring person‐centered care and strengthening investment in primary healthcare [36].

## 4. Conclusion

In the included studies (*n* = 17), conducted across diverse geographical and cultural contexts (Brazil, Colombia, Trinidad and Tobago, Canada, England, and Israel), the low frequency and lack of proactivity of physicians and nurses in addressing sexuality with older adults are notable. Healthcare professionals often adopt a pathological view of aging and sexuality, predominantly associated with genitality and largely detached from dimensions of pleasure, psychosocial, and cultural factors. In most cases, they intervene only when prompted by patients, primarily offering a therapeutic approach. Ageist beliefs also influence HIV and AIDS prevention, contributing to delayed diagnoses and suboptimal care. The main barriers to discussing sexuality include lack of time, fear of embarrassing patients, and discomfort related to insufficient training.

Sexual health requires a broad understanding of sexuality and increased investment in both initial and continuing education for healthcare professionals [1, 11, 32, 33]. The included studies highlight the importance of providing care in a welcoming environment characterized by privacy, sufficient time, and inclusive language, where information about sexuality is shared and older adults’ beliefs on the topic are appropriately addressed. They also suggest the need to extend interventions beyond clinical settings, involving the media in the dissemination of positive messages about sexuality and aging.

Study limitations include the restriction of the search to open‐access articles, which may have introduced selection bias by excluding relevant studies published in subscription‐based journals, including potentially high‐quality peer‐reviewed evidence. This approach may have limited the scope and representativeness of the review findings. Additionally, the inclusion of more comprehensive grey literature databases, such as OpenGrey or ProQuest Dissertations, could have strengthened efforts to minimize publication bias, although their exclusion was a considered decision by the authors. Another limitation is the exclusion of studies conducted exclusively with older LGBT individuals or institutionalized populations, which may have limited the understanding of sexual health promotion interventions implemented with these groups.

However, the lack of attention to diversity and aging in the daily practices of healthcare professionals further reinforces the need for research into their experiences, practices, and perceptions regarding discussions of sexuality with older adults. This is particularly relevant in Portugal, where no studies on this topic were identified, strengthening the need for primary research on professionals’ perspectives and practices. Such research could contribute to improving the quality of care provided, identifying existing constraints, and highlighting potential areas for improvement. These findings may also support the enhancement of both initial and continuing education for healthcare professionals, as well as the development of public policies that prioritize training, improve accessibility to these services in PHC, and promote sexual health literacy campaigns that are inclusive of diversity in sexuality and aging.

## Author Contributions

The systematic review of the literature was conducted by all authors, who read and agreed with the published version of the manuscript.

## Funding

In the case of the first author, this work was supported by National Funds through FCT—Foundation for Science and Technology, under the project UID/05198/2025—Centre for Research and Innovation in Education (inED) with DOI identifier https://doi.org/10.54499/UID/05198/2025 and https://doi.org/10.54499/UID/PRR2/05198/2025.

In the case of the third author, this work was funded by National Funds through FCT‐Foundation for Science and Technology, under Project UID/05739/2025 (Adult Education and Community Intervention Research Centre, CEAD), https://doi.org/10.54499/UID/05739/2025.

## Ethics Statement

The authors declare that they followed the protocols of their workplace regarding the publication of data.

## Conflicts of Interest

The authors declare no conflicts of interest.

## Data Availability

The data that support the findings of this study are available from the corresponding author upon request.
